# National response to the COVID-19 Omicron variant crisis in the ambulatory hemodialysis service in the State of Qatar

**DOI:** 10.5339/qmj.2022.38

**Published:** 2022-08-23

**Authors:** Abdullah I. Hamad, Muhammad Asim, Muftah A. Othman, Essa A. Abuhelaiqa, Alaedine Shurrab, Ihab T. Elmadhoun, Hassan Ali Al-Malki, Mohamad M. Alkadi

**Affiliations:** ^1^Division of Nephrology, Department of Medicine, Hamad General Hospital, Doha, Qatar E-mail: ahamad9@hamad.qa; ^2^Division of Nephrology, Department of Medicine, Hazm Mebaireek General Hospital, Doha, Qatar; ^3^Division of Nephrology, Department of Medicine, Al Khor Hospital, Al Khor, Qatar; ^4^Division of Nephrology, Department of Medicine, Al Wakra Hospital, Al Wakra, Qatar

**Keywords:** Qatar, Coronavirus, COVID-19, Omicron variant, end-stage kidney disease, hemodialysis

## Abstract

The coronavirus disease (COVID-19) pandemic has had a significant worldwide impact since its emergence in 2019. End-stage kidney disease patients have been among the most vulnerable population affected and have a higher risk of acquiring infection and developing more severe disease. We have encountered three major COVID-19 waves in Qatar and they have required different strategies to overcome. The most recent wave was due to the Omicron variant characterized by higher transmissibility. The monthly incidence of COVID-19 infection during the Omicron wave in patients with end-stage renal disease peaked at 256 patients compared to 35 and 39 patients during the first and second waves, respectively. In addition, more than one-third of our dialysis staff became infected during this wave. Unlike the previous two waves, COVID-19 due to the Omicron variant was less severe with only 5% of hemodialysis patients requiring admission to the intensive care unit compared to 25% during the previous waves. The Omicron variant wave resulted in a crisis in our country due to the high number of non-hospitalized COVID-19 hemodialysis patients and the severe staff shortage. Several measures were taken to overcome the crisis, such as designating one facility to dialyze all COVID-19 ambulatory patients, reducing dialysis sessions to 3 hours, and introducing a fourth dialysis shift.

This article describes the challenges we faced in the ambulatory hemodialysis service during the Omicron wave and the measures taken in the COVID-19 and non-COVID-19 designated facilities to combat the crisis.

## Background

Coronavirus disease (COVID-19) due to the original severe acute respiratory syndrome coronavirus 2 (SARS-CoV-2) virus and its different variants has had a significant impact worldwide. More than 500 million cases of COVID-19 have been reported and more than 6 million deaths have been recorded worldwide as of June 2022^1^. End-stage kidney disease (ESKD) patients receiving dialysis have been among the most vulnerable population affected by the COVID-19 pandemic and have had a higher risk of acquiring the infection and having a more severe disease course^2–5^. Mortality from COVID-19 infection has been among the highest in ESKD patients, with reported death rates of 15%–35%^2–5^. In response to this rising concern, recommendations have emerged to guide nephrologists and caregivers of ESKD patients to prevent COVID-19 infection^6–8^. Although these guidelines have paved the way for COVID-19 prevention and management, recent studies continue to report uncontrolled infection rates and high mortality^9–11^.

With the emergence of the SARS-CoV-2 B.1.1.529 (Omicron) variant in South Africa, the World Health Organization designated the virus as a variant of concern (VOC) due to its high contagiousness and transmissibility^12^. Furthermore, the Omicron variant has many genetic mutations in the spike proteins leading to concerns regarding the ability of the virus to evade immunity and the effectiveness of the currently available vaccines^13^. Unlike the original virus, Omicron has spread to 86 countries within 3 weeks of its emergence^15^. Qatar reported the first four cases due to the Omicron variant on December 17, 2021^16^. Since then, daily COVID-19 cases in the general population continued to rise and peaked at 4,169 cases per day in less than 4 weeks, with Omicron being the dominant variant in the country^1^.

There was high transmissibility in dialysis patients (likely related to frequent use of transportation, proximity to other patients for prolonged periods, and difficulty adhering to general prevention roles, such as social distancing and masking, on routine and regular bases). The observation that the Omicron variant results in less disease severity led to a crisis in the ambulatory hemodialysis (HD) service, particularly since a significant number of dialysis patients and staff became infected within a short time but the majority did not require hospitalization. Different countries managed dialysis patients differently during the Omicron epidemic. In this study, we summarize the effect of the Omicron variant on HD patients and staff and how it resulted in a crisis in the ambulatory HD service in Qatar. We also report the national response and protocols developed by the nephrology crisis committee to combat the crisis.

## Ambulatory Hemodialysis Service in Qatar

Qatar rests on the western coast of the Arabian Gulf. It has an area of nearly 11,600 km^2^ and a population of approximately 2.79 million^17^. The high prevalence of diabetes mellitus, hypertension, heart disease, and obesity among the citizens and migrants has translated into an increased ESKD burden^18,19^. Analysis of annual renal data from Qatar in 2021 revealed that the prevalence of ESKD patients treated with renal replacement therapy was 737 per million population; 44.5% on HD, 12% on peritoneal dialysis, and 43.5% with a functioning kidney allograft.

**Figure 1.**
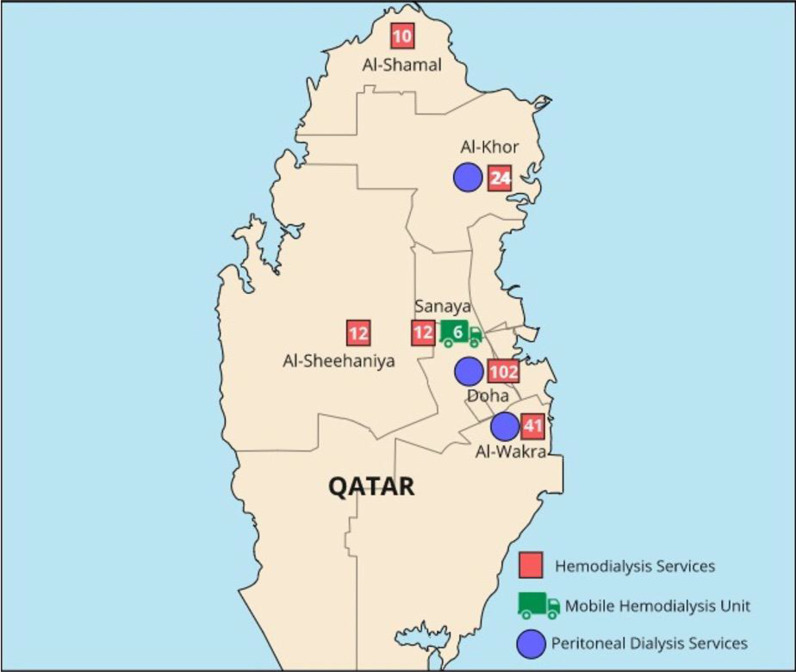
Geographical distribution of outpatient dialysis facilities in Qatar; the numbers in the red boxes represent the number of hemodialysis stations at that center.

The ambulatory HD facilities conform to the population density distribution in the country, as shown in Figure 1. Each HD unit runs at least two shifts daily, 6 days a week. Most patients are dialyzed three times weekly for at least 4 hours per session. On-site trained nephrologists supervise the treatment and follow uniform care processes and treatment guidelines that homogenize dialysis patient care in all centers. A small number of patients receive nurse-assisted home HD treatment.

## Crisis in the Ambulatory Hemodialysis Service during the COVID-19 Omicron Surge

**Figure 2.**
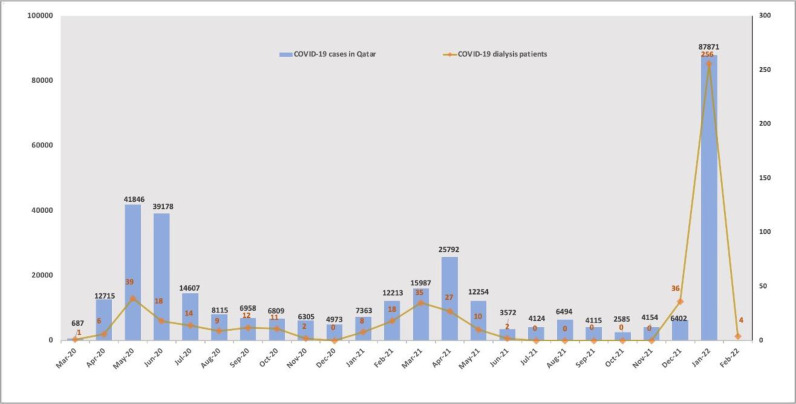
COVID-19 waves in the general population (blue bars) and dialysis patients (orange line) in the State of Qatar.

**Figure 3.**
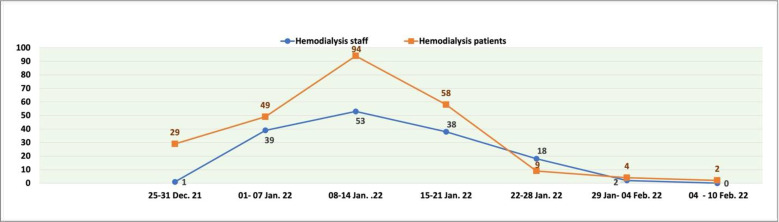
Weekly new cases of COVID-19 in hemodialysis patients (orange line) and staff during the Omicron variant wave.

**Figure 4.**
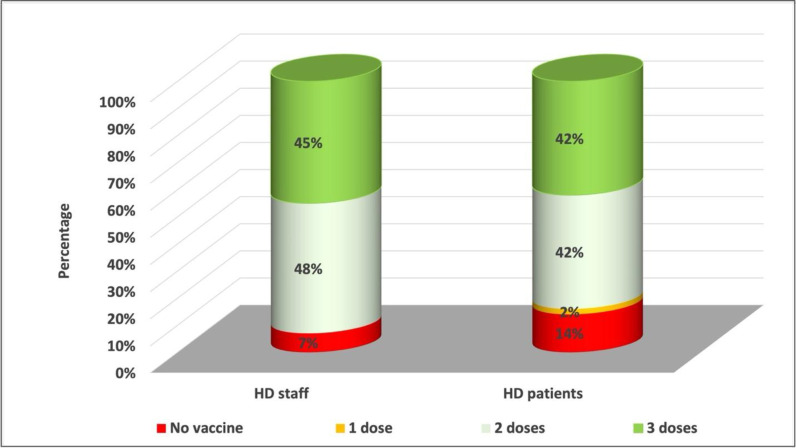
Immunization status of hemodialysis patients and staff who contracted COVID-19

Compared to the original COVID-19 virus and the Delta variant, the Omicron variant resulted in less disease severity and a higher peak during a shorter period (weeks vs. months) (Figure 2). COVID-19 cases due to the Omicron variant started in our dialysis patients and staff during the last week of December 2021. A surge in cases occurred among patients and staff over the next 2 weeks and reached its peak during the second week of January 2022. Then, there was a rapid decline in cases over the next few weeks, as shown in Figure 3. Overall, 151 of 350 HD staff (43%) and 245 of 1,020 patients (24%) tested positive for COVID-19. The high infection rate in staff compared to patients was partly due to common transportation offered by the hospital and social gatherings during the holiday season. Interestingly, more staff than patients had received at least two doses of an mRNA vaccine before their COVID-19 infection, as shown in Figure 4 (228/245 [93%] vs. 127/151 [84%] p = 0.004). Overall, the severity of illness was mild to moderate among staff, and none required hospitalization. However, 42% of the patients (103) required hospitalization. Most of the patients stayed for less than 48 hours as a precaution; 12 patients (5%) required intensive care unit admission, and five patients (2%) died. Twenty-three patients (9%) had a previous COVID-19 infection.

In the previous COVID-19 waves, all dialysis patients were hospitalized for close monitoring given their higher risk of severe disease compared to the general population. Among the dialysis patients, 72% developed pneumonia, 25% required admission to the intensive care unit, and 15% died during the first wave of COVID-19 in Qatar^3^. The Omicron variant led to a crisis in the ambulatory HD service, as most COVID-19 patients did not require hospitalization, and infected HD nurses and technicians were not permitted to rejoin work for at least 7 days. Thus, during this crisis, we had to dialyze many non-hospitalized COVID-19 HD patients, isolate COVID-19 patients from other non-COVID-19 HD patients, and manage the severe workforce shortage in our HD units.

## Ambulatory Hemodialysis Service Response to the Omicron Crisis

### Administrative

Soon after announcing the first four cases of the Omicron variant in the State of Qatar in December 2021, a nephrology crisis committee was formed under the leadership of the chief of nephrology at Hamad Medical Corporation, responding promptly to the challenge. The committee consisted of medical directors of the dialysis units, the director of dialysis nurses (the overall lead dialysis nurse), the dialysis head nurses, and chief dialysis technicians, representing all hospitals and ambulatory dialysis units. The committee met weekly, more often if needed, to get updates from different facilities and take timely actions. Having such a committee enabled designating of one unit to dialyze all COVID-19 ambulatory HD patients. It also allowed redistributing the staff and resources, as needed, to cover different facilities during the crisis. Additionally, there were daily updates from all facilities regarding the number of available dialysis slots and the number of patients and staff who tested positive for COVID-19 to determine workforce needs at each site and provide immediate support.

The committee made several important decisions during the Omicron crisis, such as canceling annual leave of the staff and asking all staff on leave to return to work (unless they had an emergency or sick leave). We also mobilized nurses from quality, research, and specialty programs, such as anemia and mineral bone disease, to join the HD nursing workforce. In addition, we increased the nursing: patient ratio from a baseline ratio of 1:2 to 1:3, and we increased working hours from 8 to 12. Moreover, we reduced the duration of the dialysis sessions to 3 hours, if a patient's condition allowed, and increased the number of dialysis shifts in COVID-19 designated units to four shifts to meet the increasing number of infected, non-hospitalized HD patients. Although we temporarily suspended routine monthly patient reviews, we maintained monthly laboratory investigations, monitored critical values, and addressed all of the HD patients’ needs throughout the Omicron crisis to ensure that the standard of care was maintained. These measures helped overcome the severe staff shortage and allowed us to mobilize staff across different facilities without affecting the patient standard of care or safety with proper monitoring and observation. A new ten-station dialysis unit was prepared as a backup plan, anticipating a rise in infected dialysis patients. Thankfully, due to the brief duration of the crisis, the measures taken were sufficient and we did not need to use the new unit. The crisis committee decisions are summarized in Table 1.

### Covid-19 Facility Response

The Ministry of Health had designated Hazm Mebaireek General Hospital (HMGH) as the main COVID-19 facility for every COVID-19 wave in Qatar. Therefore, we positioned a six-station mobile dialysis unit there and dedicated it to COVID-19 patients since the first wave started. We also had another 12-station dialysis unit at HMGH that was only used for non-COVID-19 ESKD patients.

**Figure 5.**
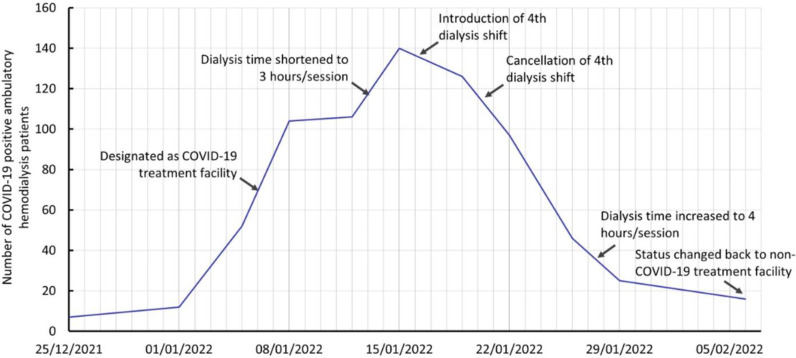
Timeline of the steps taken at the COVID-19 designated ambulatory hemodialysis unit (HMGH) during the Omicron crisis

The Omicron wave caused a surge in the number of infected home-quarantined HD patients, and the six-station mobile unit reached its maximum capacity of 36 patients within 10 days. Then, we converted the 12-station dialysis unit into a COVID-19 designated unit and transferred all non-COVID HD patients (n = 45) to other dialysis units. Moreover, we converted one of the HMGH rooms into a temporary three-station dialysis room to accommodate the rising number of COVID-19 ambulatory HD patients. We also shortened the time (by updating treatment and the discharge pathway) to transfer patients from HMGH back to their non-COVID dialysis units using time-based criteria (10 days from the first positive test) instead of waiting 21 days for a negative PCR test result. Despite all efforts, we reached the maximum capacity of 126 dialysis patients (21 dialysis stations ×  3 shifts), so we temporarily reduced dialysis time from 4 to 3 hours and started a fourth shift of dialysis. The steps taken at the HMGH facility to accommodate the Omicron wave against time and the number of positive cases on dialysis are summarized in Figure 5.

### Non-covid-19 Facilities Response

During the Omicron crisis, we implemented several measures in the non-COVID-19 ambulatory dialysis units to capture HD patients with COVID-19 before spreading the infection among other dialysis patients. For example, temperature and Ehteraz, a local smartphone application that shows if a patient should be in quarantine due to a COVID-19 infection, was checked by security at the entrance of the dialysis units. We also limited the number of companions, enforced social distancing, and transferred all meetings to virtual instead of physical to limit gatherings.

**Figure 6.**
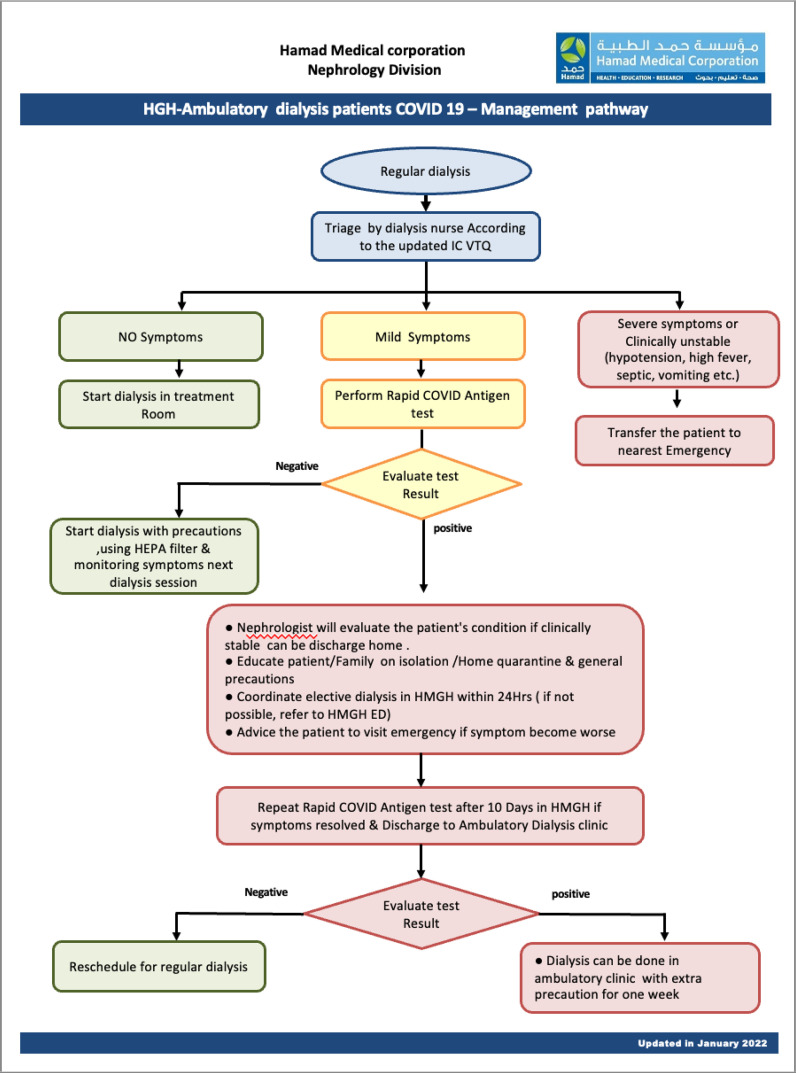
Pathway to triage HD patients and transfer them from non-COVID-19 dialysis units to COVID-19 designated dialysis units and vice versa. IC-VTQ: infection control visual triage questionnaire (a simple local triage tool that focuses on symptoms, exposure, and the presence of fever)

We used a screening questionnaire for patients before initiating dialysis. A rapid antigen COVID-19 test was performed in suspected cases and positive patients were directed to HMGH for dialysis, as shown in Figure 6. During this wave, the government allowed transportation using patients’ private cars instead of restricting transportation using an ambulance as in previous waves.

Once patients returned from the COVID-19 facility, we used extra precautions (full PPE, private or isolation rooms, or at the corner of shared rooms, use of high-efficiency particulate air filters when available).

## Conclusions

COVID-19 due to the Omicron variant has resulted in a crisis in the ambulatory dialysis service in the state of Qatar. This VOC led to a surge in the number of infected, non-hospitalized HD patients and a severe shortage in the HD workforce. Here we report a unique experience of combating the Omicron wave from a Middle East country. Several preemptive steps were taken to provide optimal care for COVID-19 patients while retaining non-COVID-19 patient safety and well-being. Leadership efforts to redistribute resources and the workforce averted a bad outcome during this wave.

### Acknowledgments

We profusely thank Dham T, Joseph J, Aly S, Singh P, Yasin F, Akl T, Ibrahim R, and all the Hamad Medical Corporation staff for their devotion to patient care during the COVID-19 pandemic. We also thank Hamza Asim for the computer graphics.

## Figures and Tables

**Table 1:** Summary of the nephrology crisis committee decisions during the COVID-19 Omicron wave

**First meeting January 2, 2022**• Appoint a lead dialysis nurse and a lead dialysis technician for all dialysis units.• Generate daily census of HD patients and staff with COVID-19.• Dialyze all COVID-19 patients at one facility (HMGH).• Support the HD staff at the COVID-19 designated facility (HMGH) by mobilizing nurses and technicians from other dialysis units.

**Second meeting January 4, 2022**• Change nurse to patient ratio from 1:2 to 1:3.• Reduce dialysis hours to 3 hours unless clinically indicated.• Convert the 12-station unit to a COVID-19 designated unit and transfer all non-COVID-19 patients (n = 45) to other dialysis units.• Transfer infected HD patients from HMGH back to their dialysis units after 10 days from the first positive test rather than waiting until their PCR is negative.

**Third meeting January 11, 2022**• Increase staff working hours from 8 to 12 hours.• Suspend monthly patient's review; however, keep monthly blood investigations and review of critical values.• Develop a pathway for positive patients’ referral from non-COVID19 dialysis units to HMGH.

**Fourth meeting January 18, 2022**• Start a fourth dialysis shift at HMGH.• Implement the newly developed pathway for referring COVD-19 patients’ to HMGH.• Prepare a new 10-dialysis station unit and designate it to COVID-19 cases.

**Fifth meeting January 25, 2022**• Go back to 4-hours dialysis sessions.• Go back to three shifts at HMGH.• Transfer back patients with COVID-19 to their dialysis units at day 7 if asymptomatic.• Resume monthly patients reviews.

